# WASP: the World Archives of Species Perception

**DOI:** 10.1093/database/baad003

**Published:** 2023-02-28

**Authors:** Tuan Nguyen, Robert Malina, Ilias Mokas, Antonis Papakonstantinou, Orestes Polyzos, Maarten P M Vanhove

**Affiliations:** Research Group Environmental Economics, Centre for Environmental Sciences, Hasselt University, Martelarenlaan 42, Hasselt 3500, Belgium; Research Group Zoology, Biodiversity and Toxicology, Centre for Environmental Sciences, Hasselt University, Agoralaan gebouw D, Diepenbeek 3590, Belgium; Research Group Environmental Economics, Centre for Environmental Sciences, Hasselt University, Martelarenlaan 42, Hasselt 3500, Belgium; Research Group Environmental Economics, Centre for Environmental Sciences, Hasselt University, Martelarenlaan 42, Hasselt 3500, Belgium; Upstream, Kastorias 4, Gerakas, 15344, Athens, Greece; Upstream, Kastorias 4, Gerakas, 15344, Athens, Greece; Research Group Zoology, Biodiversity and Toxicology, Centre for Environmental Sciences, Hasselt University, Agoralaan gebouw D, Diepenbeek 3590, Belgium

## Abstract

While human perception can play a role in influencing public support for species conservation, the mechanisms underlying human perception remain poorly understood. Some previous studies on perception have focused on a few specific taxa, which makes the understanding of the public perception of species at large a resource- and time-intensive task. Here, we introduce the World Archives of Species Perception project that consists of an animal survey and a plant survey to construct the first systematic database to study the human perception of the floral and faunal diversity at a global scale. We provide a description of our survey method, species selection, survey implementation and a discussion of the potential uses of our databases in multidisciplinary research. In the animal survey, we cover 1980 International Union for Conservation of Nature (IUCN)–evaluated species, representing 25 classes, 192 orders, 1037 families and 1705 genera. In the plant survey, we cover 2000 IUCN-evaluated species, representing 13 classes, 93 orders, 386 families and 1968 genera. Data from the survey will be collected and made available 24 months after the publication of the article.

**Database URL** http://wasp-project.net/

## Background

### Public perception and species conservation

The perception of humans toward nature is a concept extensively studied in the field of environmental psychology. For example, the ‘biophilia hypothesis’ by Kellert and Wilson (1) states that people have an innate connection to nature and that positive feelings (‘biophilia’) and negative feelings (‘biophobia’) are the products of a complex, adaptive, bio-cultural learning process that is linked to the early humans’ experience with nature and continuously built up over time. The biophilia theory sets the foundation for several research branches exploring human perception of species, particularly looking at humans’ basic emotions manifesting from the perception of specific species or species groups, which can either be positive (happiness and surprise) or negative (fear, disgust, anger and sadness) (2, 3). Recent studies found empirical evidence that the emotions toward species are among the underlying factors that influence people’s contribution to species conservation (4, 5). Thus, human perception collectively (i.e. public perception) represents the general view of society and can influence public contribution to species conservation in many ways, including environmental attitude (2), public awareness and attention for species (6, 7), willingness to fund conservation (4), organizational choice of species to promote (8, 9) or protect (10, 11) and targeted contribution to species research and knowledge (12–14). Despite such implications, little effort has been dedicated to further understand the extent to which public perception influences species conservation, which suggests that this area is in urgent need of research.

Among the concepts relating to the human perception of species, ‘species charisma’ has been one of the most extensively researched and relevant topics in recent decades, and it also illustrates the possible impacts of human perception in species conservation. The ‘charisma’ of species can be defined as the ‘distinguishing properties (of a species) that determine its perception by humans and its subsequent evaluation’ (15). Thus, species charisma is humans’ subjective appreciation toward a particular species, which may be but is not necessarily shared across cultures or through time (16).

Nature conservation organizations traditionally utilize charismatic species in promotional campaigns as an effective instrument to attract public affection and funding resources (17). One prominent example is the logo of World Wildlife Fund for Nature, portraying the charismatic giant panda that has become a global emblem of conservation and helped the organization gain massive public support since its release in 1961 (18). However, despite the mediatic, practical and economic advantages, targeted promotion of charismatic species is a highly controversial approach (for an extensive discussion, see (19)). One widespread line of criticism argues that such an approach may further enhance the ever-present perception bias among scientists, conservation organizations and the general public toward large vertebrates (20) because most charismatic species communicated to the public belong to a tiny fraction of biodiversity, particularly large-bodied mammals and birds (8, 9). Targeted promotion of those few privileged species also diverts public attention and hence public funding resources toward species possessing those particular traits, which may trigger public favoritism for charismatic species over primary conservation considerations, such as species’ endangered status (21) or ecological importance (22–24). Hence, another line of criticism questions the use of species ‘charisma’—a purely subjective concept by definition—over other objective, ecologically functional qualities such as ‘indicator’, ‘umbrella’ or ‘keystone’ species in species communication and promotion (19). For instance, the case of butterfly conservation under the European Habitat Directive (25) illustrates a contrasting example where conservation prioritizing charismatic species may exclude those of ecological importance and conservation priority. Such situations are common when the species’ charisma and ecological importance are non-overlapping.

**Figure 1.**
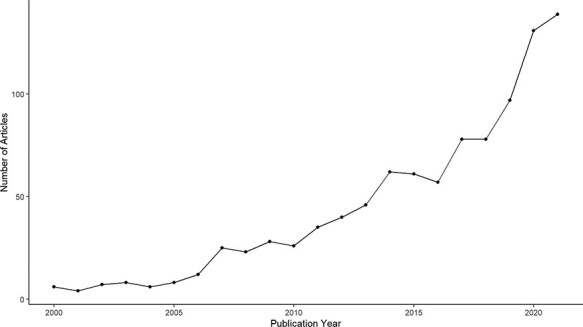
Annual publication of research articles containing the keyword ‘charismatic species’ between 2000 and 2021, extracted from Clarivate Web of Science.

Nevertheless, scientific attention toward charismatic species has been growing in recent decades, emphasizing the role of species charisma in conservation science (Figure 1). Rather than excluding the use of species’ charms, conservation scientists are studying how to use charisma more effectively by combining with other functional measures directly linked to biodiversity, such as place-based species richness (26), species’ importance for their ecosystem (27) or the presence of apex predators in some specific habitats (28). In some cases, charismatic species can also possess ‘indicator’, ‘umbrella’ or ‘keystone’ qualities (e.g. the African bush elephant *Loxodonta africana*), making the conservation of these species both publicly supported and beneficial to many other species sharing the same habitats. Yet, such cases are limited and conservation primarily focusing on these species will inadvertently forsake a wide diversity of species that do not live in the same habitat (29). Thus, a major limiting factor to the potential of charisma in biodiversity conservation lies in the root bias associated with how species charisma is often portrayed in conservation communication: ‘charismatic species mean essentially some large birds and mammals’ (19). In a quantitative review, Berti *et al.* (30) combine data from nine published studies that looked at the popularity of >13 000 vertebrate animals among humans and agreed that body size is a good proxy for vertebrate charisma. Yet, if body size is the sole determining factor of charisma across the entire animal and plant kingdoms, then the use of species charisma directly implies an impasse for conservation scientists and practitioners in trying to reduce taxonomic bias in species promotion simply because the majority of species are small-sized. Interestingly, recent studies have revealed quantitative evidence that species charisma is multidimensional and the charisma-defining features are largely dependent on the context. For instance, in a study of Australian birds, Garnett *et al.* (31) employed a stated preference survey and found that respondents prefer small, colorful and melodious birds to large ones, implying that body size is not always the determining trait of charisma in birds. Contradictorily, the same study also found that when asked to directly nominate the most attractive birds, the same respondents instead tend to name large birds that are more commonly presented in the Australian cultural context. The difference in response, as explained by the authors, could be because of the higher public knowledge of the large birds due to them being more popularly promoted in the Australian society. Such biased promotion implies a feedback loop that continuously favors some charismatic species at the exclusion of the others.

In another context related to the rarely mentioned crossover between the study of charisma and invertebrate animals, Salvador *et al.* (32) showed that the public is highly interested in this megadiverse group of species and that science communication should be improved to provide knowledge matching the public preference. After a decade and a half of species charisma research, it is becoming apparent that conservation scientists and practitioners understand proportionately better the charisma of mammals (33, 34) than other vertebrates such as birds (31) and fishes (35) and invertebrates (32). No studies have explored the charisma of species in the plant kingdom. The case of species charisma signifies the necessity to advance our scientific understanding of the public perception of species charisma toward a wide range of species, including the understudied vertebrate, invertebrate and plant groups. Existing literature indicates that there are potentially more and diverse groups of charismatic species beyond what we currently know, and thus, expanding the scientific knowledge of species charisma opens up additional venues for effective species communication and conservation.

While the understanding of species charisma is a merit in itself, the need to improve the scientific understanding of how the public perceives species in general is not less relevant. This is because, on one hand, positive, biophilic emotions such as the affection for charismatic species can lead to conservation actions, but, on the other hand, negative, biophobic emotions such as fear and disgust can also lead to disconnectedness and reduced willingness to conserve nature (4, 5) or worse, such as species culling and eradication (36). Thus, the knowledge of human perception of species is crucial for conservation organizations to facilitate species communication programs that are attractive and more taxonomically inclusive; raise awareness and potentially reattribute public attention and funding toward lesser-known species; expand the collection of species and species traits that are appealing to the public or identify alternative aspects to promote species often associated with prejudices and negative emotions such as snakes, spiders or insects.

### The necessity for a broad-scaled public perception database

A major challenge in studying human perception of species is that no global database on the public perception of animal or plant species exists. Regarding animals, previous efforts have resulted in scattered datasets looking at specific vertebrate taxa, including a focus on mammals and birds in charisma studies (30, 33, 34), with the rare exception of butterflies (25, 37) and a focus on few specific reptile and invertebrate taxa such as snakes and spiders in biophobia studies (3, 32, 38, 39). These previous studies do not represent all feasible manifestations of human appreciation or alertness for faunal diversity, let alone provide indications for the perception-defining traits throughout the animal kingdom. Furthermore, existing studies have employed various techniques to measure perception, ranging from web data extraction to surveys, which have been shown to be incompatible (31).

The case of human perception of the plant kingdom is similar to that of invertebrates—our scientific knowledge of the human perception of plants, including plant charisma, is scarce. The few existing studies that have explored this topic have suggested two distinguishable research pathways, either looking at individual species traits or at plant communities forming an esthetical landscape. In the former direction, the focus is on the individual plant level—the researcher identifies the plant features that elicit humans’ response. Research in this path is often interested in species with visual esthetics, such as large, tall, long-lived trees (40) or colorful, spectacular flowers (41, 42). This topic is mainly studied in tourism research for its practical implications for the tourism sector (40, 42). In the latter direction, the focus is on public satisfaction when viewing a landscape that consists of groups of one or several plant species among other non-biological features, which has direct implications in urban landscape planning (43–45). To the best of our knowledge, no detailed studies exist on the public perception of plants and its implications for conservation. This may be partly associated with the well-documented ‘plant blindness’, a perception bias that leads to plant conservation receiving significantly less scientific attention and fewer funding resources than vertebrates (12, 46).

Still, we argue that the connection between the public perception of plants and nature conservation is important for at least four reasons. First, the conservation of plants is urgent because plants include some of the most abundant species on the Earth and are crucial for the functioning of ecosystems (47), whereas a major part of plant diversity is threatened or at risk of extinction (48). Second and most importantly, humans have a long history of coevolution and cohabitation with plants (49). Thus, plants play an irreplaceable role in the socio-economic and cultural development of human societies. Through generations of localizations and adaptations, human societies have developed vastly distinct local knowledge and perception of native floral diversity. In many instances, the diverse interactions between human cultures and plant communities can give rise to the discovery of unique and endemic forms of knowledge, or ‘indigenous knowledge’, which further enhances the ways in which humankind perceives nature as a whole (50). The knowledge of plants reflects how humans perceive plants’ values in general and is extensively useful in a diverse range of applications in the form of provisional and cultural ecosystem services, including food, material, biomedical applications, and esthetical and cultural values (51). However, modern human society causes rapidly increasing disconnectedness from nature (52), which erodes humans’ cultural knowledge and collective memory of species (53). As Jarić *et al.* (53) explained, this phenomenon, which is referred to as the ‘societal extinction of species’, can weaken pro-environmental attitude and behavior, leading to degraded support for species conservation. Third, the concept of ‘charismatic plant’ has been extensively used in species promotion and raising awareness—including as national symbols, by conservation organizations (such as botanical gardens, natural parks and conservation science) and plant-related businesses (plant nurseries, seed companies, florists, etc.)—suggesting the applicability of the knowledge of human perception toward plants in a real-world context. Fourth, many established links between the diversity of plants and the conservation of other biota, such as animals, require an understanding of how humans relate to the concerned plants. For instance, certain plants help maintain biodiversity in anthropogenic settings (54, 55) or define conservation-relevant natural biomes (56, 57).

### Aims of the WASP Project

In the ‘World Archives of Species Perception’ (WASP) project, we aim to fill the gap of knowledge on the human perception of species by constructing two separate global databases for animals and plants. The ambition of the WASP project is 3-fold: (i) to initiate a holistic investigation of the public perception across a large sample of the visible biodiversity, (ii) to provide scientists with a means to study how public perception can be linked to species’ traits and how it can help facilitate more effective conservation and (iii) to compare public perception across different species groups to identify the discrepancies between current scientific communication and public interest.

We build on an existing citizen-science approach, which has recently become a fertile ground for producing large-scale biological databases [e.g. iNaturalist, Global Biodiversity Information Facility (GBIF), Bird Sounds Global and PlantNet] and is well endorsed by conservation scientists and ecologists (13, 20). We choose to implement the survey using the contingent rating method, visualized in the format of six simple and uniform questions for each species, which potentially allows the respondents to easily understand the questions presented and answer them quickly. This design is intended to capture respondents’ intuitive feelings that elicit realistic, rather than rational, respondents’ choice behavior (58). Previous survey studies have often limited the investigation to a few hundred species due to cost constraints, which hinders sufficient response collection (3, 34, 38). By simplifying the survey design, we expect to encourage higher numbers of responses per respondent and per species, which enables adequate statistical power to study species samples containing thousands of species, and at minimal economic costs. We launch the survey on a web platform in order to increase the reach and ease of access of the WASP surveys across multiple online and offline channels. Our approach aims at collecting perception data at the global level. Hence, instead of imposing demographic constraints, we encourage as many responses as possible from the general public. This approach can be suitable to systematically and holistically study the public perception of species over a large sample of participants and across a wide diversity of animal and plant species. To the best of our knowledge, this is the first database to apply a choice modeling survey to elicit human perception across a wide range of animal and plant groups.

In the WASP surveys, we limit our scope to the species evaluated under the International Union for Conservation of Nature’s (IUCN) Red List, in order to link the public perception of species with their conservation status. The IUCN Red List represents one of the most comprehensive lists of species’ population and conservation information to date (59). In each survey, we draw a sample species list, taking into account the species evaluated under the IUCN Red List and having at least one image available on iNaturalist, using a stratified random sampling strategy that maximizes species’ taxonomic distinctiveness in our sample (see Species Selection and Sampling Strategy). In total, our database for animals (WASP-A) contains the public perception of 1980 animal species, organized into 10 groups as used by the IUCN Red List (mammals, birds, fishes, reptiles, amphibians, insects, mollusks, crustaceans, corals and other invertebrates) in terms of six traits: cuteness, dangerousness, beauty, intelligence, endangered status and importance for ecosystem. Our database for botany (WASP-B) includes the public perception of 2000 species representing five groups (algae and mosses, ferns and allies, conifers, cycads and monocots and dicots) in terms of six traits: familiarity, impressiveness, beauty, endangered status, importance for humans and importance for the ecosystem. The choices of traits are discussed in Trait Selection and Trait Scales.

The remainder of this article is organized as follows. In Survey Design and Implementation, we explain in detail the survey design and implementation of the WASP surveys. In Database Use and Discussion, we highlight some hypotheses, database usability and limitations.

## Survey design and implementation

**Figure 2.**
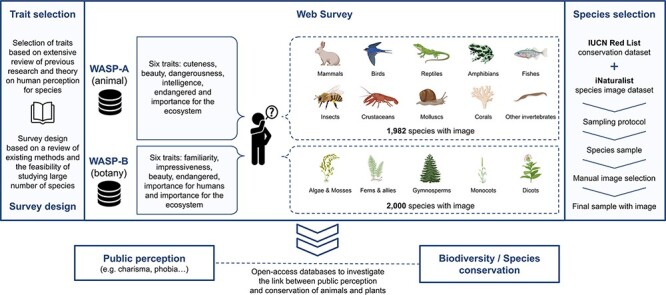
Schematic overview of the WASP surveys.

Figure 2 presents a schematic overview of the procedure we used to construct the WASP surveys. The choice of survey method (Survey Method) and species traits (Trait Selection and Trait Scales) are based on an extensive review of previous public perception literature. We described the species datasets that we used (see Species Datasets) and conducted a sampling strategy to select the species sample for each WASP survey (Species Selection and Sampling Strategy). We then manually matched a qualified image from iNaturalist for each species in the sample and provided adjustments where necessary (Image Selection). The complete survey was implemented via a web-based interface, with method described in Web Survey Application.

### Survey method

Known public perception studies to date have employed two broad classes of methods: web data extraction methods and survey methods. In the web data extraction (‘proxy’) methods, public perception such as species charisma is proxied through the occurrence frequency of search terms on various Internet sources, such as Google Web Search (6, 37, 60), Wikipedia (61) and online social media (e.g. Facebook and Twitter) (62, 63), which is cost-efficient to implement and can cover a broad range of species. A major downside of this approach is that the proxied measure of public perception can be biased toward species that are more frequently promoted, rather than more appealing to the public (31). As a result, the proxy methods cannot accurately measure the public interest associated with the species’ esthetic features that are linked to their physical, perceivable traits. In the survey methods, biases can be controlled for by directly asking respondents specific questions, which means that the measure of public perception can be more accurate (23, 39, 64–66). Stated preference methods, particularly the class of methods called choice modeling, are a powerful and informative group of survey methods (67). They allow the researcher to identify significant stimuli that affect human perception by categorizing species based on a predefined set of species traits (called ‘attributes’) with different levels, such as species’ body sizes, morphological features or evolutionary distinctiveness, and designing the alternatives for optimal information extraction even at relatively low sample size (68). Previous studies on the public perception of species employed various stated preference methods, including contingent valuation (24), discrete choice experiment (22, 31) and pairwise comparison (34, 35). However, applying these methods often requires creating hypothetical trade-off scenarios (in contingent valuation); predetermining a set of species features (in choice experiment) or completing a high-dimensional comparison matrix to an adequate statistical power (in pairwise comparison (69)), which can be resource-intensive, can be difficult to determine or can generate researcher bias. Such requirements could severely restrict the range of species to be studied, which would limit the exploratory potential of the WASP databases.

In order to construct databases that are representative and comparable among a diverse range of animal and ‘plant’ groups, we made efforts to avoid technical assumptions associated with the survey methods as described earlier. We chose to implement the contingent rating method from the class of choice modeling (67). This survey method enabled us to create a design that is more suitable for the aims of the WASP project than other survey methods, by utilizing three features. First, the contingent rating method directly requests the respondents’ stated preference for each alternative (that is, species) instead of the changes in respondents’ preference across multiple alternatives. This feature removes the requirements associated with multi-alternative survey methods, such as the need to predefine the set of attributes in a choice experiment or the need to adequately complete the comparison matrix in a pairwise comparison. Second, each questionnaire consists of six similar, simple and uniform questions across all alternatives, which can potentially encourage more choices from the respondents within their survey time in comparison to complex questionnaires. These two features, direct preference and simple questionnaire, help alleviate the burden of containing a wide range of animal and plant species in the WASP surveys, which would otherwise be resource-intensive using other survey methods. The use of simple questions is crucial in this case because they elicit intuitive responses in the form of feelings rather than reflective responses in the form of rational, deductive processes (58), the former of which is tightly linked to human perception, especially esthetically (15). Third, the contingent rating method is compatible with a diverse range of statistical analysis techniques, including the random utility framework (67), which enables *post hoc* analyses of public perception to a level as refined as the species’ traits.

The choice of our approach also takes into consideration the general limitations of the stated preference methods, including reliability (i.e. do people answer honestly) and representativeness (i.e. external validity) (50). With respect to reliability, we only ask intuitive questions while ensuring the anonymity of the respondents, which reduces the risk of the respondents feeling pressured into giving strategic responses. We attempt to account for the representativeness of the databases by spreading the survey widely and keep it open indefinitely in hope of collecting a significant number of responses over time.

**Figure 3.**
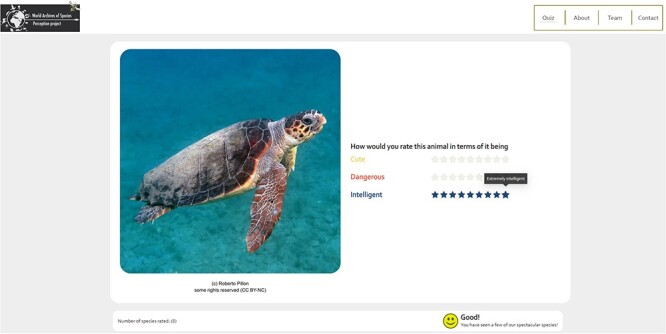
Snapshot of the WASP web survey.

The WASP surveys are implemented in the form of web surveys and contain three major parts: (i) introductory pages, which present general information about the WASP project and an instruction for taking either of the WASP surveys; (ii) the WASP-A web survey, which presents the public perception questionnaire for animal species and (iii) the WASP-B web survey, which shows the public perception questionnaire for ‘plant’ species. On each survey webpage, an image of a randomly selected species from our sample is shown and the respondent is asked to give a rating for each trait listed (Figure 3).

We used a nine-point Likert scale, with the score of 1 indicating either the negative extreme of a bipolar trait or the least agreement to a 1D trait and a score of 9 implying either the positive extreme of a bipolar trait or the most agreement to a 1D trait (for a complete list of traits, see Trait Selection and Trait Scales). To further reduce potential mental constraints, we present the six traits in pairs of three on each webpage. A response of one species is recorded in our database only when all six traits have been rated. After all traits are rated for the first species, a consent form is presented in accordance with the General Data Protection Regulation to provide necessary information to the respondents before they can officially participate in the survey. Upon receiving consent, the web survey automatically loads the image of the second species and the respondents can continue with the rating process. A nudge consists of the total number of species rated, and an emoji expressing increasingly positive emotion is included as an extra incentive for the respondents (Figure 3). The survey ends at the discretion of the respondent or when all species in the collection have been rated.

In addition to the primary variables, with consent from the respondents, we also collect their public IP addresses and cookies, which are fully anonymized given that we do not collect any demographic information from the respondents. This supplementary information is used for identifying the number of choices per anonymous respondent as well as clustering the respondents into broad geographical regions, such as at national or continental level.

### Trait selection and trait scales

In both WASP surveys, we used the existing literature related to the human perception of species reviewed earlier to identify the potential traits that have been extensively theorized or studied. Since there is no standardized set of species traits that drive public perception, for the sake of comparability across species groups, we reasoned the use of our traits by linking them to basic human emotions, such as positive emotions (happiness and surprise) and negative emotions (fear and disgust) and justifying them on the grounds of the theory of biophilia and biophobia (1). We excluded anger and sadness because these emotions are more often associated with behavior rather than species esthetics (2).

#### WASP-A: animals

In the animal survey (WASP-A), we selected six traits: (i)] cuteness, (ii) dangerousness, (iii) beauty, (iv) intelligence, (v) endangered status and (vi) importance for the ecosystem (Table 1). Cuteness and beauty represent two popular esthetic traits influencing public affection from the conservation science literature that focuses on mammals and birds (15, 33, 34, 70). We are interested in whether these traits are also perceived among species in the understudied groups and whether they contribute to the charisma or phobia of these species. Cuteness and beauty are distinct traits: the former may suggest the tendency for close physical contact with the species (cuddly) or keeping distance (disgusting), while the latter describes the esthetical impressiveness (beautiful or ugly) of the species. Some people consider the deep sea blobfish *Psychrolutes marcidus* (Actinopterygii: Scorpaeniformes: Psychrolutidae) to be ‘ugly cute’ (https://www.nationalgeographic.com/science/article/animals-ugly-cute-psychology), suggesting the two traits can be perceived independently. Thus, we assigned a bipolar scale for cuteness (disgusting–cute) and beauty (ugly–beautiful).

**Table 1.** Trait selection and trait scales

Trait name	Rating scale	Dimension
*WASP-A*		
1. Cuteness	(1) extremely disgusting–(9) extremely cute	Bipolar
2. Dangerousness	(1) not dangerous–(9) extremely dangerous	1D
3. Beauty	(1) extremely ugly–(9) extremely beautiful	Bipolar
4. Intelligence	(1) not intelligent–(9) extremely intelligent	Bipolar
5. Endangered	(1) common–(9) extinct in the wild	Other^a^
6. Importance for ecosystem	(1) extremely harmful–(9) extremely important	Bipolar
*WASP-B*		
1. Familiarity	(1) not familiar–(9) extremely familiar	1D
2. Impressiveness	(1) not impressive–(9) extremely impressive	1D
3. Beauty	(1) extremely ugly–(9) extremely beautiful	Bipolar
4. Endangered	(1) common–(9) extinct in the wild	Other^a^
5. Importance for humans	(1) extremely harmful–(9) extremely important	Bipolar
6. Importance for ecosystem	(1) extremely harmful–(9) extremely important	Bipolar

Note. The rating scale represents the two extremes of each trait. A rating score of 1 represents either the extreme negative value or the lack of perception of the trait, and a rating score of 9 represents either the extreme positive value or the full perception of the trait, depending on the dimension of the trait. Traits can be 1D or 2D (bipolar). For example, in the bipolar trait ‘Beauty’, 1 is ‘extremely ugly’ and 9 is ‘extremely beautiful’.

a The rating scale for the ‘Endangered’ trait is adjustable to reflect the levels in the IUCN Red List categories. The levels are as follows: (1) common/least concern, (2) near threatened, (3) vulnerable, (5) endangered, (7) critically endangered and (9) extinct in the wild.

Dangerousness is another trait that is frequently studied in the contexts of charisma and phobia. This may be partly due to the high correlation of beauty and dangerousness that is present in some of the most charismatic species groups we know, such as the carnivores (33, 34), and also partly due to the strong visceral feelings of fear and disgust induced by some particular species groups, such as snakes or spiders (38, 39, 64). Dangerousness is represented in a 1D scale (not dangerous–dangerous).

Anthropomorphism, or attributing human-like characteristics to species, is another popular aspect in public perception theories (71). Chan (72) discussed three species’ qualities as the strongest scientifically validated traits as the basis for humans’ empathetic anthropomorphizing of animal species, namely ‘prosociality’, ‘intelligence’ and ‘ability to suffer’. Here, we selected the trait ‘intelligence’ because it is a trait that the public can perceive for a wide range of species by looking at their images in our survey, potentially through established belief or conjecture based on the belief about the intelligence of more familiar species. ‘Prosociality’ and ‘ability to suffer’, on the other hand, are unlikely perceivable by the general public without having a prior knowledge of the species-on-display’s behavior, such as caring for offspring or responding to pain. Previous research indicates that the general public may proxy species’ intelligence through their anthropomorphic features, such as forward-facing eyes, brain size or behaviors (19, 70). However, such research has not extended beyond the scope of mammal species, making this survey among the first in collecting human perception of species intelligence across several animal taxa. We used a 1D scale (not intelligent–intelligent) for this trait.

Previous research suggests that the conservation status of mammals can influence the public preference for these species when it is shown alongside species images (34). Here, we are interested in capturing the perceived conservation status of species—a novelty of the WASP databases. This quantitative measure can help researchers study the interaction between perceived conservation status and esthetics of species and compare it with the actual conservation status, which could reveal additional insights for improving conservation communication. For conservation status, we used a 1D pseudo scale (common–extinct in the wild), which can be converted into six categories of the IUCN Red List, as follows: 1 (least concern), 2 (near threatened), 3 (vulnerable), 5 (endangered), 7 (critically endangered) and 9 (extinct in the wild). To avoid conversion errors, we made this guideline apparent for the respondents on the survey webpage.

Finally, we attempted to capture the ecological values the public perceive from species. This novel quantity allows researchers to compare species’ actual and perceived role in the ecosystem as well as to verify whether there exist systematic patterns between the public perception of species’ esthetics and species’ perceived importance across different species groups. For instance, respondents’ familiarity with a species and its role in the ecosystem has been found to correlate positively with willingness to pay for conservation in a wide range of species groups (24). For this trait, we used a bipolar scale (harmful–important) to capture both negative and positive perceptions of a species’ role in the ecosystem. Put together, we selected four traits (cuteness, dangerousness, beauty and intelligence) as potential public perception drivers and two traits (endangered status and importance for ecosystem) describing the perceived ecological role or conservation status of species.

#### WASP-B: botany

In the ‘botanical’ survey (WASP-B), we adopted the following six traits: (i) familiarity, (ii) impressiveness, (iii) beauty, (iv) endangered status, (v) importance for humans and (vi) importance for the ecosystem (Table 1). For simplicity, we refer to the species included in the WASP-B survey as plants or ‘plants’ interchangeably, as we also included a small number of species of algae (see Species Datasets).

A mechanistic difference between the human perception of animals and plants could be grounded in the fact that plants mainly consist of sessile and modular organisms. The sessility of plants renders them unable to instantaneously shift place in response to stimuli, which results in them generally being perceived by humans as inactive and without intelligence. Thus, traits that are suggestive of behavioral patterns in animals like cuteness, dangerousness and intelligence are unlikely to be perceived in the case of plants among the general public (although we do acknowledge that the notion of plant’s intelligence is subject to debate among botanists (73)). On the other hand, the abundance and unthreatening presence of plants allow humans to adopt and explore various ways to utilize them for their own needs, leading to the inseparable perception of plants for their use values for humans (51). The modular structure of a plant makes it practically challenging to distinguish among plant individuals or compare between them in a way similar to animals. This difficulty is further enhanced as plants often live within plant communities that can be both dense and taxonomically diverse, whereas the organs among plants in a community can be visually superficially similar (green leaves, brown trunks, etc.). A number of scientific studies have verified this perception bias. For instance, Hoyle *et al.* (43) found that humans often use colors—a characteristic that does not translate into species richness, as a cue to assess species diversity in a floral landscape—while Adamo *et al.* (12) found that plant scientists’ attentions are skewed toward colorful and morphologically distinct wild flowering plants. Therefore, recognition of an individual plant species often requires prior experience with the patterns inherent in the different organs of the species, such as leaf, flower, fruit or stem, which may inevitably be an emergent challenge to the general public, especially in the context of increasing disconnectedness from nature in current society (52).

The difference in public perception of plants versus animals led us to replace three traits considered for animals (cuteness, dangerousness and intelligence) with familiarity, impressiveness and importance for humans. Familiarity measures how familiar the plant is to the respondent, expressed in a 1D scale (not familiar–extremely familiar). The degree of familiarity with a species has been previously studied to influence conservation attitude (24) and can be an especially useful conservation tool for less popular groups of species (74). In our database, the familiarity measure can potentially allow the researcher to distinguish between plant enthusiasts and non-enthusiasts or relate to the level of plant blindness across respondents, geographic regions or taxa.

We chose impressiveness as another trait alongside beauty associated with ‘plant’ esthetics. In a similar reasoning to the inclusion of cuteness and beauty in the WASP-A survey, we were interested in whether the perception of plant esthetics correlates with the perception of their conservation status and role in the ecosystem. It is possible that impressiveness and beauty may be correlated in some way, such as in the case of animals (33). For plants, we expected that the two traits are not redundant but rather only partially overlapping and possibly complementary. Some plants are considered impressive but not beautiful, whereas others are beautiful but unimpressive. For example, giant elephant ear *Colocasia gigantea* (Araceae) or tumbleweed (e.g. *Kali tragus*, Amaranthaceae) is impressive because of either the gigantic leaf size or the ‘tumbling’ behavior, but both are very common and unlikely to be regarded as beautiful by local communities where they occur. Alternatively, many flowering plants, such as several species from the sunflower family (Asteraceae) like common dandelion (*Taraxacum officinale*), common sunflower (*Helianthus annuus*) or cornflower (*Centaurea cyanus*), are regarded as beautiful but unlikely to be regarded as impressive. Some other plants grab massive public attention, such as corpse flower *Amorphophallus titanum* (Araceae) in botanical gardens around the world or the mega-flora giant sequoia *Sequoiadendron giganteum* (Cupressaceae) in American national parks. While there is still a lack of studies of the public perception of charismatic plants, it is apparent that many species have been promoted for reasons other than just beauty (e.g. Meise Botanic Garden, Belgium (https://www.plantentuinmeise.be/en/pQe0zFX/masterpieces-under-glass); Kew Royal Botanic Gardens, the UK (https://www.kew.org/read-and-watch/extraordinary-plants-at-kew)). In any case, beauty can add an additional layer of affection for impressive plant species, such as in the case of the globally popular Japanese cherry blossoms *Prunus serrulata* (Rosaceae), albeit consisting of several human-bred cultivars.

Finally, we included importance for humans because humans perceive the use values of plants, as has been discussed throughout this paper. This trait is interesting on its own because it provides a collective view of how the general public perceives the value of different ‘plant’ groups, in contrast to the well-documented human-use values of thousands of plants (75). It also provides a unique opportunity to correlate this trait with other traits considered in the WASP-B survey or to other plant characteristics to identify bias-deriving perception patterns in the public, such as whether phenotypically ‘weed-like’ species are more likely perceived as harmful for humans. Altogether, we included three esthetic traits (familiarity, impressiveness and beauty) and three traits (endangered status, importance for humans and importance for ecosystem) describing the human-perceived conservation status of species and their roles for humans and nature.

### Species datasets

The IUCN Red List of Threatened Species dataset contains the conservation assessment of roughly 85 000 animal species and 60 000 plant species (59). We used this dataset as a starting point to collect quantifiable information about species’ conservation status. Next, we compared this list with the iNaturalist Research-grade Observations dataset (76), available from the GBIF to filter the species whose images are available. iNaturalist is a citizen-science web platform where organismal observations are shared by the interested community. iNaturalist hosts >40 million observation records with images of animal and plant species and has become an increasingly popular database for biological studies over the last decade (76).

**Figure 4.**
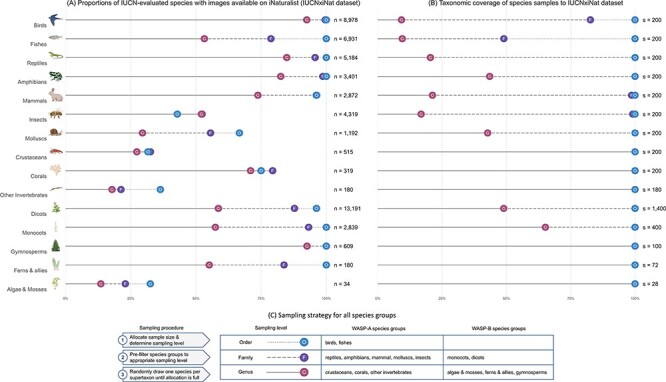
(A) Summary statistics of the IUCNxiNat species dataset presenting the proportions of species evaluated under the IUCN Red List with images available on iNaturalist (left). (B) Taxonomic coverage of the species sample resulting from our sampling strategy to the IUCNxiNat dataset (right). (C) Sampling strategy chosen for all species groups in WASP-A and WASP-B surveys (bottom). The *y*-axis represents the species groups, each marked with an illustrative icon. The *x*-axis represents a measure of proportion, or coverage, in percentage. Values are expressed in terms of various taxonomic ranks, including genus (denoted as G with solid line), family (denoted as F with dashed line) and order (denoted as O with dotted line) levels.

We match the two datasets using species’ scientific names because we found that this method performs slightly better than using the recommended GBIF Taxonomic Backbone (77) in terms of the total number of matched species between the two datasets. The matching task returned lists of 33 881 (43%) IUCN-evaluated animal species and 16 853 (25%) IUCN-evaluated plant species. We used the resulting matched list of species, hereby referred to as the ‘IUCNxiNat’ species dataset, as the base species dataset for our sampling procedure. The datasets retain a relatively high taxonomic coverage of species evaluated under the IUCN protocol for vertebrates and vascular plants, while the taxonomic representativeness for most invertebrate groups and algae and mosses is limited (Figure 4A). The species group with the lowest match in WASP-A is other invertebrates (18% genera covered) and in WASP-B is algae and mosses (14% genera covered). The low taxonomic coverage in these groups is partially attributed to the fact that nine invertebrate groups (Collembola, Chilopoda, Maxillopoda, Ostracoda, Polyplacophora, Monoplacophora, Hexanauplia, Polychaeta, Turbellaria and Enopla) in WASP-A and six algae and moss groups (Anthocerotophyta, Sphagnopsida, Takakiopsida, Chlorophyceae, Ulvophyceae and Florideophyceae and Polytrichaceae) in WASP-B are not covered in our species datasets because IUCN-evaluated species from these groups do not have research-grade images available from iNaturalist.

### Species selection and sampling strategy

In each of the two WASP databases, we aim to survey a total of 2000 species. In WASP-A, we evenly distributed the species allocation to 10 major species groups defined in the IUCN Red List: mammals, birds, fishes, amphibians, reptiles, insects, mollusks, crustaceans, corals and other invertebrates [containing arachnids (Arachnida), sea urchins (Echinoidea), sea cucumbers (Holothuroidea), millipedes (Diplopoda), annelids (Clitellata), velvet worms (Udeonychophora), starfishes (Asteroidea) and horseshoe crabs (Merostomata)] to allow cross-comparisons across groups. In other words, 200 species were sampled within each major IUCN-defined group of animals. We chose to assign an equal sample to each major animal group in trying to facilitate a balance between the biased human interest for vertebrate species and the overwhelming natural species richness in invertebrate species (20, 59).

In WASP-B, we chose to stratify sample size relatively proportional to the number of IUCN-evaluated species in each group. This approach helps adequately representing the phenotypic variation among the more species-rich vascular plant taxa while ensuring that the non-flowering plant groups are not under-represented in our database. We categorized the species into five groups, adapted from the IUCN Red List categorization, with the number of allocated species in brackets: algae and mosses (28), ferns and allies (72), gymnosperms (100), monocots (400), and dicots (1400). Algae and mosses are grouped to simplify the sampling procedure, given that there are only 34 species belonging to these groups in our species dataset.

An ideal sample should include species allocations such that the phenotypic (in this case, morphological, as perceived based on an image) variation across all sampled species is maximized. How the public assesses the morphology of organisms relates to esthetics, which is a subjective element. Moreover, there is no standard approach applicable across all organism groups for the researcher to quantify morphology a priori. In some specific contexts, manual selection of species by comparing species images on a case-by-case basis may be an option, where the number of species studied is small and the public is familiar with the taxonomic group (e.g. mammals (34)), but such an approach is prone to emphasizing bias when looking at the whole animal or plant kingdom. Thus, and despite the nuances that taxa of the same rank are scarcely comparable in such classification (78), we chose to use taxonomic distinctiveness, represented by the taxonomic classification system used by the IUCN Red List, as the main proxy to sample species.

Our sampling procedure aimed to maximize the number of taxa across all taxon ranks. In particular, we conducted three different sampling variations to achieve this objective. In the first variation, for species groups that contain few species and genera in our filtered dataset (see Species Datasets), we grouped the species by genus and iteratively random sampled one representative species in each taxon until the allocated sample size was reached. This variation applies to crustaceans, corals and other invertebrates (WASP-A) and also to algae and mosses, ferns and allies and gymnosperms (WASP-B). The second variation is conducted at one taxonomic rank higher and requires a pre-filtering step. Initially, only one random representative species is filtered for each genus. This filter helps collapse the species pool of the relevant species groups to its number of distinct genera, thus removing the heterogeneities in the species distribution at this taxonomic rank. Then, one species per family is randomly selected from this subset. This process is repeated until the number of species reaches the corresponding allocation size. This variation is conducted for mammals, reptiles, amphibians, insects and mollusks (WASP-A) and for monocots and dicots (WASP-B). In the third variation, the same procedure is applied but at one taxonomic rank higher—pre-filtering at the family level and sampling at the order level. This variation concerns birds and fishes (WASP-A), which are the two classes that have more distinct families in the species dataset than our allocated sample size.

To summarize, our sampling strategy follows a three-step procedure: (i) allocate species sample size for each species group; (ii) collapse the species dataset to an appropriate sampling level, which is the highest sampleable taxonomic rank given allocation size and (iii) sampling one species for each taxon at one taxonomic rank higher and repeat this process until the sample size is reached (Figure 4C). In WASP-A, this sampling strategy results in the final animal collection containing 1980 species (only 180 species in the group other invertebrates in our species dataset), spanning 25 classes, 192 orders, 1037 families and 1705 genera. In WASP-B, we obtain a plant collection of 2000 species, covering 13 classes, 93 orders, 386 families and 1968 genera. The sampling procedure returns lists of species that are relatively representative of the species dataset, fully covering taxa down to the family level, with the exception of birds and fishes due to the high number of families in these groups (Figure 4B).

### Image selection

#### Animal images

**Figure 5.**
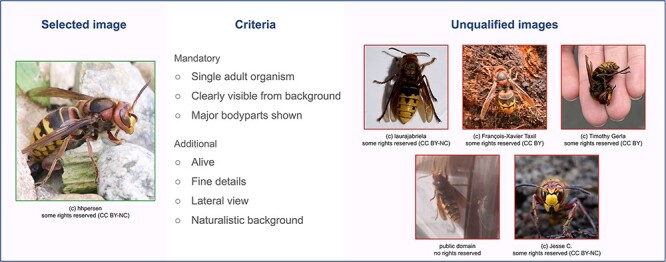
Image selection criteria.

For each species in the WASP-A sample, we manually select the most appropriate image by browsing through the available images of this species on iNaturalist. We limit the search only to images with research-grade quality and a Creative Commons license. We create a set of selection criteria that help in ensuring that our species image gallery is relatively homogeneous in quality and clearly displays the details of the species (Figure 5). The most appropriate images are selected following a two-step selection. First, we filter a number of potential image candidates that satisfy all of the mandatory criteria. Then, the candidate meeting the highest number of additional criteria is manually chosen for the WASP gallery. Extra measures regarding image quality were found to help reduce image bias (34). For species for which a potential image candidate is not found, a replacement species is re-sampled from a subset of species with similar conservation status and being taxonomically closest to the removed species.

#### Plant images

The strategy devised for selecting animal images cannot be applied in the context of plants. As previously discussed, the human perception of plants exists at different scales, such as modular, individual or landscape, which makes it difficult to determine a single image that could represent the public perception of a particular plant species. In addition, plant morphological features are sensitive to seasonal variations, such as the timing of abscission or flowering. Such temporal patterns further diversify the esthetics of plants and add to the challenge of representing a ‘plant’ esthetic by a single image. Failure to account for the diverse perception-triggers can lead to deficient results. For example, an image of the giant sequoia *S. giganteum* (Cupressaceae) containing only the cone and leaf parts is expected to trigger a less intense perception than an image capturing the view of the whole tree due to the impression the public holds for its massive size. A potential solution is to contain as many potential triggers as possible, for instance, by having separate images of plant parts as well as its entire habitus. Investigating this issue requires extended time and resources, which we will document in detail in a future update.

### Web survey application

The development of the WASP web survey entails three components: (i) the front-end (web) application, (ii) the back-end (web) application and (iii) the database.

The front-end (web) application is a responsive, mobile-first single-page application built in React.js. and is divided into four layers: (i) the application programming interface (API) layer, which is responsible for all API operations, error handling and endpoint definitions; (ii) the context layer, which acts as a centralized data store and exposes modification functions to the various components; (iii) the components and assets layer, where all the component implementations reside, alongside all the static assets (images, icons and fonts) and (iv) the styles layer, which is written in SCSS (Sassy CSS), a cascading style sheet (CSS) precompiler.

The API back-end application is written in JAVA and is built on the SpringBoot framework. In particular, the structure follows the package-by-feature approach, which groups feature-related classes in the same package. This monolithic approach leads to an application with high cohesion and modularity and minimal coupling between packages (see Appendix B).

**Figure 6.**
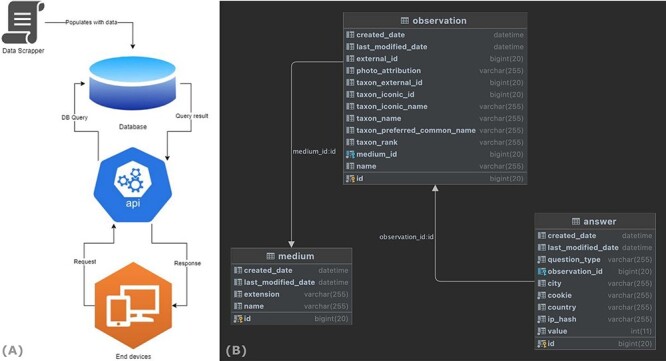
End-to-end data flow of the WASP web surveys.

The back-end application serves as a public API server and is used by the front-end application to display the data and track user interactions. Moreover, this application is also responsible for database transactions and data persistence. For example, when a user browses the application, a data flow is initiated by a request to the server (Figure 6A). This request is then consumed by the back-end application and forwarded to the database server as a database query. The query result is then pushed to the back-end application, where it is converted to a JavaScript object notation (JSON) response, before it is sent back to the web application. A simplified end-to-end data flow is shown in Figure 6A.

The application data are persisted in a MySQL database (Figure 6B) and include three tables: (i) the ‘medium’ table, which holds all the metadata about the images; (ii) the ‘observation’ table, which holds the information related to each animal species (including a medium id) and (iii) the ‘answer’ table, which stores all the data related to each user’s response.

## Database use and discussion

### Database usability

**Figure 7.**
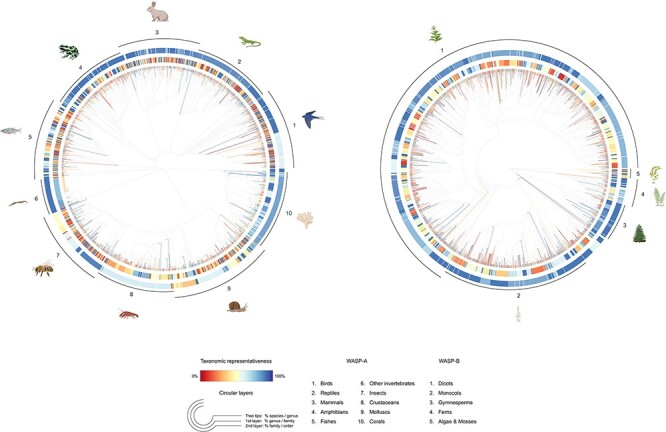
Tree-like structure illustrating the taxonomic representativeness of the WASP-A (left) and WASP-B (right) species samples. Color scale explains the taxonomic representativeness (in percentage covered) compared to the IUCN Red List species. Colored tree tips show the percentage of species covered in our species sample, by genus; the first (inner) and second (outer) circular layers represent the genus-coverage by family and family-coverage by order, respectively. The synthetic tree–like structure was generated from the Open Tree of Life, using the R package ‘rotl’.

The WASP databases contribute a novel and systematic way for researchers to explore the public perception of a diverse range of animal and ‘plant’ species. The animal and ‘plant’ samples we have selected represent ∼58% and ∼76% of all species families evaluated under the IUCN Red List for these groups, respectively (Figure 7). The complete lists of our species samples are available (see Supporting Information), as well as a summary statistics table (Appendix A, Table A1) and a table illustrating the species composition of our samples by the most and least frequently selected taxa (Appendix A, Table A2).

Studying the databases can improve the current state of knowledge of public perception in conservation; for instance, by revealing how the public generally perceives species belonging to different taxonomic groups; identifying whether some species groups are more associated with negative emotions than others, which may hinder conservation efforts; discovering additional, potentially charismatic species or verifying empirically existing theories relating to public perception of species. As we have discussed throughout this paper, the knowledge gained from the databases can help conservation managers make strategies align more closely with both public interests and conservation objectives.

The WASP databases can be used to study perception at the regional scale, by grouping respondents based on geographic location. We expect that the public perception can be vastly different across regions, such as in the case of public preference for the most favorite charismatic mammals (34). This regional heterogeneity creates an opportunity for researchers to link species promotion strategies with public perception and, to a lesser extent, evaluate the effectiveness of previous strategies on public awareness.

The scope of the WASP project extends beyond the perception of the species on display by potentially providing predictive insights for a wide range of species. One prominent approach to predicting the human perception of a given taxon is applying machine learning to species images. In this approach, a first set of species images is used to train a machine learning model to develop and detect graphical patterns that influence the variables of interest; the trained model is then used to predict the variables of interest for other species image datasets. This approach has recently been employed to evaluate the esthetic value of species at a global scale, with a special focus on reef fishes (35). The WASP species collection and image gallery allow our selection of images to be treated as training materials for the prediction of public perception over a diverse range of animal and other groups.

Although we emphasize our focus on pioneering a holistic investigation of the public perception of animals and ‘plants’ and its implications for conservation science, our WASP databases can be directly beneficial to related cross-disciplines and interdisciplinary research. Environmental psychologists, for instance, are interested in understanding the mechanisms underlying human emotions for wildlife and the implications on environmental behavior and attitude (4, 5). In environmental economics, public perception motivates the progression of economic theory toward the realization that the value of species goes beyond materialistic needs; for example, by recognizing the cultural, bequest and existence values people place on these species (23, 24) or the supporting value arising from their functional roles in ecosystems (27). It has been argued that reconciling the utilitarian and non-utilitarian views better reflects the basis of human nature: humans are neither purely materialistic nor spiritualistic (70). Indeed, each individual person can have a sophisticated value system, reflected by our worldviews and knowledge, which guides us in perceiving the instrumental, intrinsic and relational values of nature differently (50). In support of interdisciplinary research, the WASP databases are designed to be readily compatible with other species’ datasets. We adopt both the taxonomic information system used by the IUCN Red List and the species’ unique identifier from GBIF, making it simple to merge databases using species identity such as scientific names, unique identifiers or the GBIF Backbone Taxonomy (77).

### Limitations and future directions

Given the exploratory scope of the WASP databases in collecting public perception of species, here, we discuss the limitations and prospects of our approach. Our choice of traits allows for a diverse range of exploratory research questions on public perception, including species charisma and phobia. Noteworthily, although these traits were carefully selected based on an extensive list of literature, they do not constitute a definitive list of traits perceptible by humans. At best, they should only be seen as a set of pioneering aspects initiating the first comprehensive studies of public perception toward diverse groups of plants and animals. As the database expands and the knowledge of public perception broadens over time, more traits and species can be easily added to the databases in future updates, owing to the flexibility of our approach.

Although the number of species contained in each of our WASP databases (∼2000) is several times higher than that in previous public perception survey studies (30, 35, 38), our databases still only represent a modest fraction of the (morphological) diversity when looking at the millions of species on the planet (79). The species sample size was determined by considering the trade-off between representing species diversity and representing the perception of the public; that is, an increase in the number of species to be surveyed implies an expected fewer number of responses per species, assuming a fixed population of respondents, and vice versa. Several factors can influence this balance, including survey method and design, expected total number of responses and expected number of responses per respondent. We opted for a strategy to simplify the survey design as much as possible and aim to encourage at least 100 000 responses, or equivalently 50 responses per species, in each WASP survey. While our approach may be adequate to compare the public perception of species at the family level or higher taxonomic ranks, the restricted sample size prevents our databases from capturing the full perception spectra within lower taxonomic ranks, such as at the genus or species level. For instance, out of the 36 species from the charismatic cat family (Mammalia: Carnivora: Felidae) in the WASP-A species sample, only the Asiatic golden cat (*Catopuma temminckii*) and the Chinese mountain cat (*Felis bieti*) are present in our sample, which leaves out well-known charismatic species such as the tiger (*Panthera tigris*), lion (*Panthera leo*) or snow leopard (*Panthera uncia*). Nevertheless, we expect that the findings from the WASP databases can help researchers visualize the first public perception spectra of animal and plant species, from which additional species samples can be constructed to investigate the public perception at a more refined level.

While we used the IUCN Red List taxonomic classification scheme as a proxy for sampling species based on phenotypic variation, the observed perception outcomes are solely dependent on the respondent and the image of species on display, thus exogenous from the taxonomic classification the species was sampled from. As our species sampling approach is based on a maximization algorithm prioritizing the higher-to-lower taxonomic ranks, we ensured that the species in our samples are as taxonomically differentiated as possible. Thus, small changes in the taxonomic classification of species will not fundamentally alter the public perception mapping of our database. However, in the event that there are significant changes to the ways humans classify species biologically, extended surveys covering additional species can be added to reflect the newly generated gap of knowledge on the public perception of species groups that are insufficiently considered in our current version of the databases. While we acknowledge that scientific progress constantly adds to the body of biological information that can be taken into account in conservation, perhaps most notably thanks to advances in molecular biology (80), the relative importance of genetic and phenotypic information in the delineation of groups of organisms for conservation decisions still remain a largely debatable subject (81). As our databases focus on how people perceive organisms based on their phenotype, this is beyond the scope of our current study and the only consideration we can take in this respect is keeping our databases open and extendable.

Although the WASP databases focus on species and public perception on a global scale, we further argue that investigating public perception at a local scale is another important aspect for species conservation: while conservation communication and funding efforts spread globally, many actual conservation programs happen in a local context and involve local communities. Coincidentally, biodiversity conservation efforts can sometimes be in conflict with the developmental goals of local communities, those who are often associated with being less urbanized and more disadvantaged (82). Thus, conservation programs need to consider the perspective of local communities in developing biodiversity targets if they are to be successful (83). For example, in many cases of wild, rare medicinal plants (84–87), conservation programs that facilitate the balance between conservation and harvesting for economic uses by the local community (e.g. by commercialization/domestication of the plants or incentives to conserve) may be more attractive to local communities and lead to higher community support. The Convention on Biological Diversity realized the importance of local knowledge and placed it at the core of its strategic framework (Strategic Plan for Biodiversity 2011–2020, including Aichi Biodiversity Targets. Available at: https://www.cbd.int/sp/). Our approach can be readily adopted in this context to initiate extensive studies of the local community perception toward a diverse range of local culturally important fauna and flora.

## Supplementary Material

baad003_Supp

## Figures and Tables

**Figure A1.**
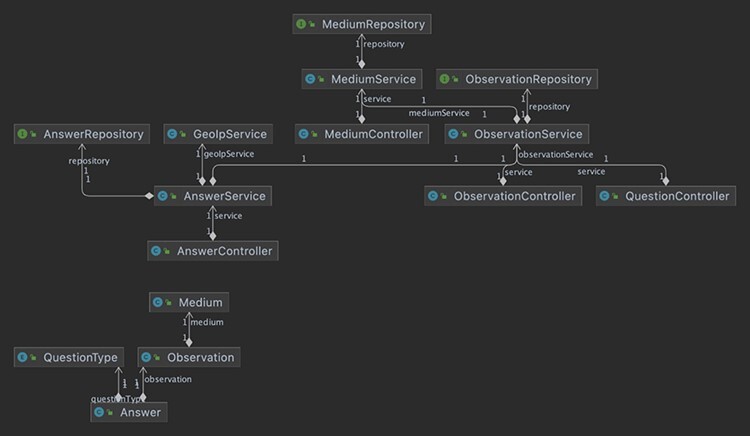
Business layer classes.

## Data Availability

We used R Statistical Software (v4.1.2; R Core Team 2021; https://www.R-project.org/) for the production of the raw version of all quantitative figures and completed using basic image editing software. Species group illustrations were downloaded from BioRender (https://biorender.com/). The synthetic tree-like structures of our species samples were generated using the Open Tree of Life R package ‘rotl’ (Michonneau, Brown and Winter 2016; doi: 10.1111/2041-210X.12593). Tree data manipulation and graphical illustration were done using the following R packages: ‘*ape*’ (Paradis and Schliep 2019; doi: 10.1093/bioinformatics/btg412) and ‘*ggtree*’ (Yu 2017; doi: 10.1002/imt2.56). The codes underlying this article will be shared on reasonable request to the corresponding author.

## Appendix A: Additional information of species samples

**Table A1.** Species samples descriptive statistics

		Species samples Mean number of species(SD)			IUCN Red List Total number of taxa(% covered by WASP samples)		
	Species group	Genus	Family	Order	Genus	Family	Order
1	Birds	1(0)	1(0)	5.56(15.72)	2380(8.40)	244(81.97)	36(100)
2	Fishes	1(0)	1(0)	3.13(2.00)	3949(5.06)	518(38.61)	64(100)
3	Reptiles	1(0.07)	2.25(0.87)	50(76.29)	1150(17.30)	93(95.70)	4(100)
4	Amphibians	1(0.16)	2.70(1.35)	66.67(76.50)	550(35.82)	75(98.67)	3(100)
5	Mammals	1(0)	1.34(0.49)	7.69(11.35)	1296(15.43)	162(91.97)	27(96)
6	Insects	1(0.07)	1.82(0.83)	22.22(20.82)	2302(8.64)	223(49.32)	21(42.86)
7	Mollusks	1(0.07)	1.49(0.52)	7.69(15.14)	1611(12.35)	243(55.14)	39(66.67)
8	Crustaceans	1.42(0.54)	7.41(7.97)	33.33(75.29)	519(27.17)	83(32.53)	19(31.58)
9	Corals	1.90(1.42)	7.41(8.40)	33.33(74.84)	148(70.94)	34(79.41)	8(75.00)
10	Other invertebrates	2.77(6.01)	6.67(13.69)	15(28.47)	365(17.81)	127(21.25)	33(36.36)
11	Dicots	1(0)	5.26(5.33)	27.45(37.78)	4896(28.59)	303(87.79)	53(96.23)
12	Monocots	1(0)	7.27(11.54)	40(41.68)	1069(37.42)	59(93.22)	10(100)
13	Gymnosperms	1.33(0.48)	8.33(10.44)	20(36.08)	81(92.59)	12(100)	5(100)
14	Ferns and allies	1.11(0.31)	2.32(1.92)	5.14(10.17)	118(55.08)	37(83.78)	14(100)
15	Algae and mosses	1(0)	1.27(0.55)	2.15(1.91)	211(13.27)	96(22.92)	40(32.50)

**Table A2.** Species sample composition, illustrated by the most and the least frequently selected orders

Major group	Four taxa with the most species, in decreasing order; one taxon with the least species (number of species in bracket)				
Birds	Passeriformes (95)	Charadriiformes (19)	Piciformes (9)	Caprimulgiformes (8)	Trogoniformes (1)
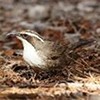
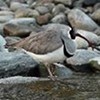
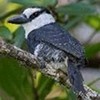
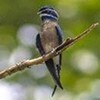
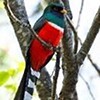
Fishes	Perciformes (8)	Beloniformes (6)	Beryciformes (6)	Characiformes (6)	Stephanoberyciformes (1)
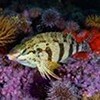
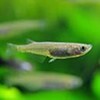
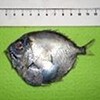
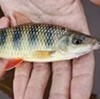
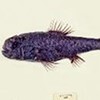
Reptiles	Squamata (163)	Testudines (29)	Crocodylia (7)	–	Rhynchocephalia (1)
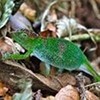
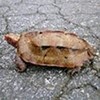
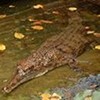
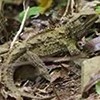
Amphibians	Anura (155)	Caudata (23)	Gymnophiona (22)	–	–
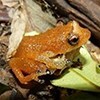
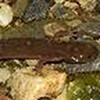
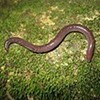
Mammals	Rodentia (45)	Cetartiodactyla (29)	Primates (25)	Carnivora (23)	Proboscidea (1)
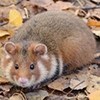
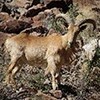
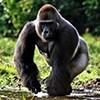
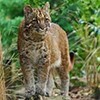
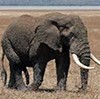
Insects	Odonata (67)	Coleoptera (41)	Orthoptera (32)	Lepidoptera (22)	Diptera (2)
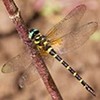
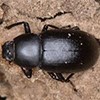
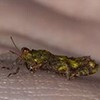
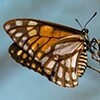
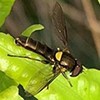
Mollusks	Stylommatophora (78)	Littorinimorpha (15)	Oegopsida (15)	Sorbeoconcha (14)	Spirulida (1)
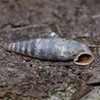
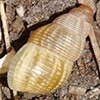
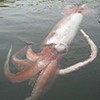
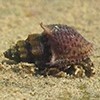
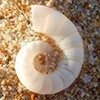
Crustaceans	Decapoda (187)	Anostraca (4)	Amphipoda (3)	Isopoda (3)	Euphausiacea (10)
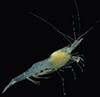
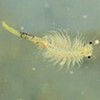
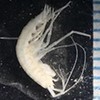
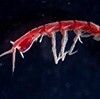
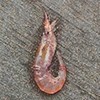
Corals	Scleractinia (188)	Actiniaria (8)	Alcyonacea (2)	Milleporina (2)	Helioporacea (1)
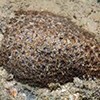
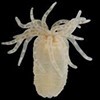
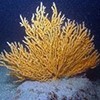
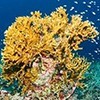
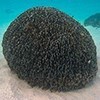
Other invertebrates	Aspidochirotida (88)	Araneae (46)	Sphaerotheriida (7)	Spirostreptida (6)	Camarodonta (1)
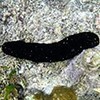
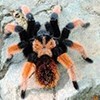
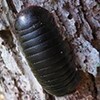
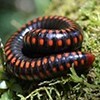
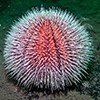
Dicots	Malpighiales (175)	Lamiales (148)	Caryophyllales (105)	Ericales (98)	Icacinales (1)
Monocots	Asparagales (113)	Poales (99)	Alismatales (77)	Arecales (44)	Acorales (1)
Gymnosperms	Pinales (84)	Cycadales (12)	Ephedrales (2)	–	Ginkgoales (1)
Ferns and allies	Polypodiales (40)	Cyatheales (7)	Salviniales (4)	Gleicheniales (3)	Psilotales (1)
Algae and mosses	Hypnales (8)	Dicranales (3)	Grimmiales (3)	Charales (2)	Porellales (1)

## Appendix B: API back-end packages

Figure A1 presents the business layer classes in the API back-end application with feature-related classes grouped into packages. These related packages are the following:

- **org.uhasselt.wasp.common** All the commonly used classes, services and interfaces reside here. This package encapsulates all the infrastructure files, configurations, error handling and logging functionalities.
- **org.uhasselt.wasp.medium** Encapsulates all the operations performed on media files.
- **org.uhasselt.wasp.observation** ‘Observation’ refers to an entity that encapsulates all the data related to one species (taxon data and medium).
- **org.uhasselt.wasp.question** Used to retrieve a random ‘Observation’ as well as the different ‘QuestionTypes’.
- **org.uhasselt.wasp.answer** Responsible for managing user answers.
- **org.uhasselt.wasp.contact** Responsible for contact via email.

All domain packages (that is, b, d and e) are further split into layers that follow a common script structure:

org.uhasselt.wasp.<package>

- controller
- domain
- repository
- service

The ‘controller’ is responsible for handling requests and responses. This is the outermost layer, as controller classes communicate with the outside world. The ‘domain’ package holds all the business model classes that define the application schema. The ‘repository’ serves as an abstraction layer between the application and the database. Specifically, this is where all the database queries and mutations take place. The database abstraction is provided by Hibernate, a JPA implementation that handles all the low-level connectivity, allowing database agnostic design. The ‘service’ serves as an intermediate layer between the controller and repository. The interaction between these layers is as follows:

- A request reaches the controller.
- The controller communicates directly with the service, passing mostly primitives derived from the request.
- The service communicates directly with the repository, using primitives and domain objects.
- The repository queries the database, converts the results to domain objects and returns these objects to the service layer.
- The service layer returns the domain objects to the controller layer.
- The controller converts the domain objects to JSON responses and responds back to the request origin.
