# Supplementary material for: WASP: the World Archives of Species Perception
Source: Database (Oxford). 2023 Feb 28;2023:baad003. doi: 10.1093/database/baad003 (PMC9972524; doi:10.1093/database/baad003)
Supplement: baad003_Supp [file baad003_supp.zip › suppl_data/Supporting Information.docx]

# Supporting Information

All of the following supporting information is available on GitHub at: https://github.com/TuanNguyen04/WASP.

**Software Use**

We used R Statistical Software (v4.1.2; R Core Team 2021) for the production of the raw version of all quantitative figures and completed using basic image editing software. Species group illustrations were downloaded from BioRender (https://biorender.com/). The synthetic tree-like structures of our species samples were generated using the Open Tree of Life R package ‘rotl’ (Michonneau, Brown and Winter 2016). Tree data manipulation and graphical illustration were done using the following R packages: ‘ape’ (Paradis and Schliep 2019) and ‘ggtree’ (Yu 2017).

**S1 Data. Sample of 1,980 species used in the WASP-A survey with image link.**

(CSV)

**S2 Data. Sample of 2,000 species used in the WASP-B survey with image link.**

(CSV)
