# Supplementary material for: WASP: the World Archives of Species Perception
Source: Database (Oxford). 2023 Feb 28;2023:baad003. doi: 10.1093/database/baad003 (PMC9972524; doi:10.1093/database/baad003)

# Appendices

**Appendix A: Additional information of species samples**

**Table A1.** Species samples descriptive statistics

**Table A1.** Species samples descriptive statistics

|  | Species group | Species samples  Mean number of species (SD) | | | IUCN Red List Total number of taxa (% covered by WASP samples) | | |
| --- | --- | --- | --- | --- | --- | --- | --- |
|  |  | Genus | Family | Order | Genus | Family | Order |
| 1 | Birds | 1 (0) | 1 (0) | 5.56 (15.72) | 2380 (8.40 %) | 244 (81.97 %) | 36 (100 %) |
| 2 | Fishes | 1 (0) | 1 (0) | 3.13 (2.00) | 3949 (5.06 %) | 518 (38.61 %) | 64 (100 %) |
| 3 | Reptiles | 1 (0.07) | 2.25 (0.87) | 50 (76.29) | 1150 (17.30 %) | 93 (95.70 %) | 4 (100 %) |
| 4 | Amphibians | 1 (0.16) | 2.70 (1.35) | 66.67 (76.50) | 550 (35.82 %) | 75 (98.67 %) | 3 (100 %) |
| 5 | Mammals | 1 (0) | 1.34 (0.49) | 7.69 (11.35) | 1296 (15.43 %) | 162 (91.97 %) | 27 (96 %) |
| 6 | Insects | 1 (0.07) | 1.82 (0.83) | 22.22 (20.82) | 2302 (8.64 %) | 223 (49.32 %) | 21 (42.86 %) |
| 7 | Molluscs | 1 (0.07) | 1.49 (0.52) | 7.69 (15.14) | 1611 (12.35 %) | 243 (55.14 %) | 39 (66.67 %) |
| 8 | Crustaceans | 1.42 (0.54) | 7.41 (7.97) | 33.33 (75.29) | 519 (27.17 %) | 83 (32.53 %) | 19 (31.58 %) |
| 9 | Corals | 1.90 (1.42) | 7.41 (8.40) | 33.33 (74.84) | 148 (70.94 %) | 34 (79.41 %) | 8 (75.00 %) |
| 10 | Other Invertebrates | 2.77 (6.01) | 6.67 (13.69) | 15 (28.47) | 365 (17.81 %) | 127 (21.25 %) | 33 (36.36 %) |
| 11 | Dicots | 1 (0) | 5.26 (5.33) | 27.45 (37.78) | 4896 (28.59 %) | 303 (87.79 %) | 53 (96.23 %) |
| 12 | Monocots | 1 (0) | 7.27 (11.54) | 40 (41.68) | 1069 (37.42 %) | 59 (93.22 %) | 10 (100 %) |
| 13 | Gymnosperms | 1.33 (0.48) | 8.33 (10.44) | 20 (36.08) | 81 (92.59 %) | 12 (100 %) | 5 (100 %) |
| 14 | Ferns & allies | 1.11 (0.31) | 2.32 (1.92) | 5.14 (10.17) | 118 (55.08 %) | 37 (83.78 %) | 14 (100 %) |
| 15 | Algae & Mosses | 1 (0) | 1.27 (0.55) | 2.15 (1.91) | 211 (13.27 %) | 96 (22.92 %) | 40 (32.50 %) |

**Table A2.** Species sample composition, illustrated by the most and the least frequently selected orders

**Table A2.** Species sample composition, illustrated by the most and the least frequently selected orders

| Major group | Four taxa with the most species, in decreasing order; one taxon with the least species (number of species in bracket) | | | | |
| --- | --- | --- | --- | --- | --- |
| Birds | 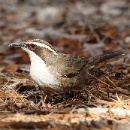  Passeriformes (95) | 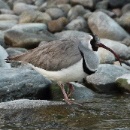  Charadriiformes (19) | 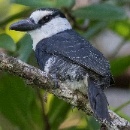  Piciformes (9) | 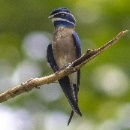  Caprimulgiformes (8) | 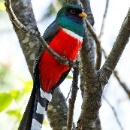  Trogoniformes (1) |
| Fishes | 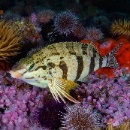  Perciformes (8) | 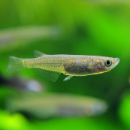  Beloniformes (6) | 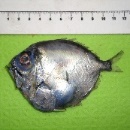  Beryciformes (6) | 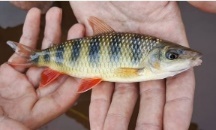  Characiformes (6) | 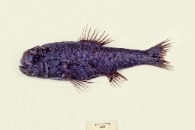  Stephanoberyciformes (1) |
| Reptiles | 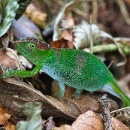  Squamata (163) | 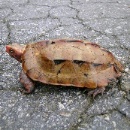  Testudines (29) | 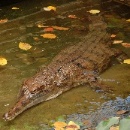  Crocodylia (7) | - | 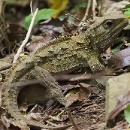  Rhynchocephalia (1) |
| Amphibians | 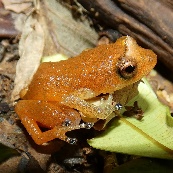  Anura (155) | 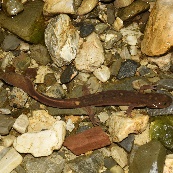  Caudata (23) | 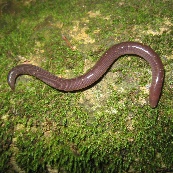  Gymnophiona (22) | - | - |
| Mammals | 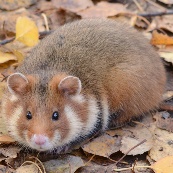  Rodentia (45) | 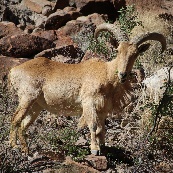  Cetartiodactyla (29) | 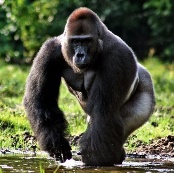  Primates (25) | 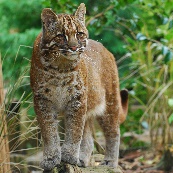  Carnivora (23) | 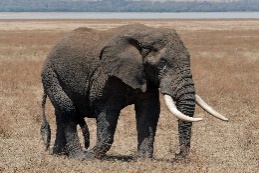  Proboscidea (1) |
| Insects | 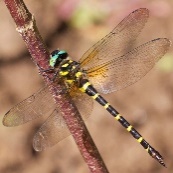  Odonata (67) | 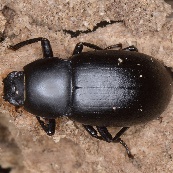  Coleoptera (41) | 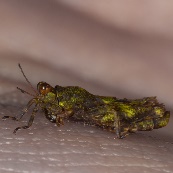  Orthoptera (32) | 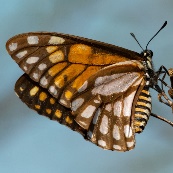  Lepidoptera (22) | 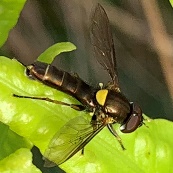  Diptera (2) |
| Molluscs | 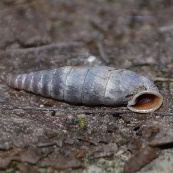  Stylommatophora (78) | 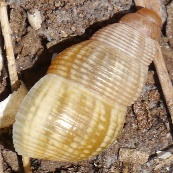  Littorinimorpha (15) | 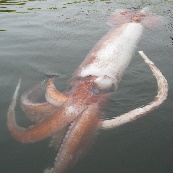  Oegopsida (15) | 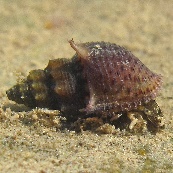  Sorbeoconcha (14) | 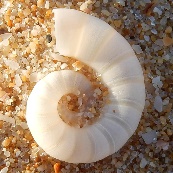  Spirulida (1) |
| Crustaceans | 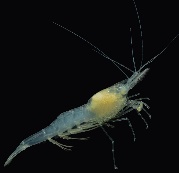  Decapoda (187) | 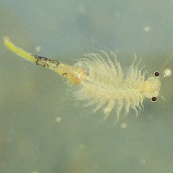  Anostraca (4) | 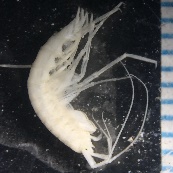  Amphipoda (3) | 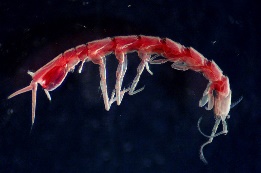  Isopoda (3) | 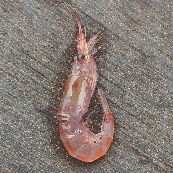  Euphasiacea (10) |
| Corals | 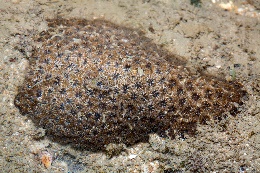  Scleractinia (188) | 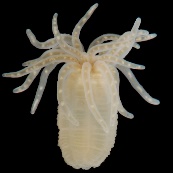  Actiniaria (8) | 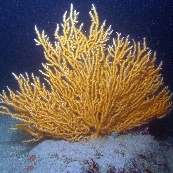  Alcyonacea (2) | 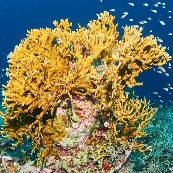  Milleporina (2) | 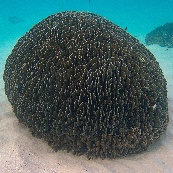  Helioporacea (1) |
| Other Invertebrates | 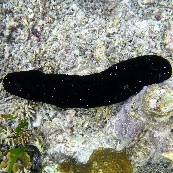  Aspidochirotida (88) | 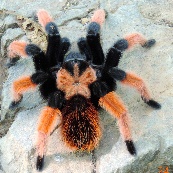  Araneae (46) | 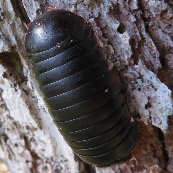  Sphaerotheriida (7) | 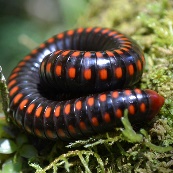  Spirostreptida (6) | 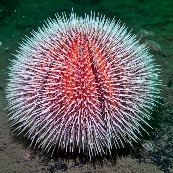  Camarodonta (1) |
| Dicots | Malpighiales (175) | Lamiales (148) | Caryophyllales (105) | Ericales (98) | Icacinales (1) |
| Monocots | Asparagales (113) | Poales (99) | Alismatales (77) | Arecales (44) | Acorales (1) |
| Gymnosperms | Pinales (84) | Cycadales (12) | Ephedrales (2) | - | Ginkgoales (1) |
| Ferns & allies | Polypodiales (40) | Cyatheales (7) | Salviniales (4) | Gleicheniales (3) | Psilotales (1) |
| Algae & Mosses | Hypnales (8) | Dicranales (3) | Grimmiales (3) | Charales (2) | Porellales (1) |

**Appendix B: API Back-end Packages**

These related packages are the following:

1. **org.uhasselt.wasp.common**All the commonly used classes, services, and interfaces reside here. This package encapsulates all the infrastructure files, configurations, error handling, and logging functionalities.
2. **org.uhasselt.wasp.medium**Encapsulates all the operations performed on media files.
3. **org.uhasselt.wasp.observation**‘Observation’ refers to an entity that encapsulates all the data related to one species (taxon data and medium)
4. **org.uhasselt.wasp.question**Used to retrieve a random ‘Observation’ as well as the different ‘QuestionTypes’
5. **org.uhasselt.wasp.answer**Responsible for managing user answers.
6. **org.uhasselt.wasp.contact**Responsible for contact via email

All domain packages (that is, b, d and e) are further split into layers that follow a common script structure:

org.uhasselt.wasp.<package>

- controller
- domain
- repository
- service

The “controller” is responsible for handling requests and responses. This is the outermost layer, as controller classes communicate with the outside world. The “domain” package holds all the business model classes that define the application schema. The “repository” serves as an abstraction layer between the application and the database. Specifically, this is where all the database queries and mutations take place. The database abstraction is provided by Hibernate, a JPA implementation that handles all the low-level connectivity, allowing database agnostic design. The “service” serves as an intermediate layer between the controller and repository. The interaction between these layers is as follows:

- A request reaches the controller
- The controller communicates directly with the service, passing mostly primitives derived from the request
- The service communicates directly with the repository, using primitives and domain objects
- The repository queries the database, converts the results to domain objects, and returns these objects to the service layer
- The service layer returns the domain objects to the controller layer
- The controller converts the domain objects to JSON responses and responds back to the request origin

**Figure A1.** Business layer classes


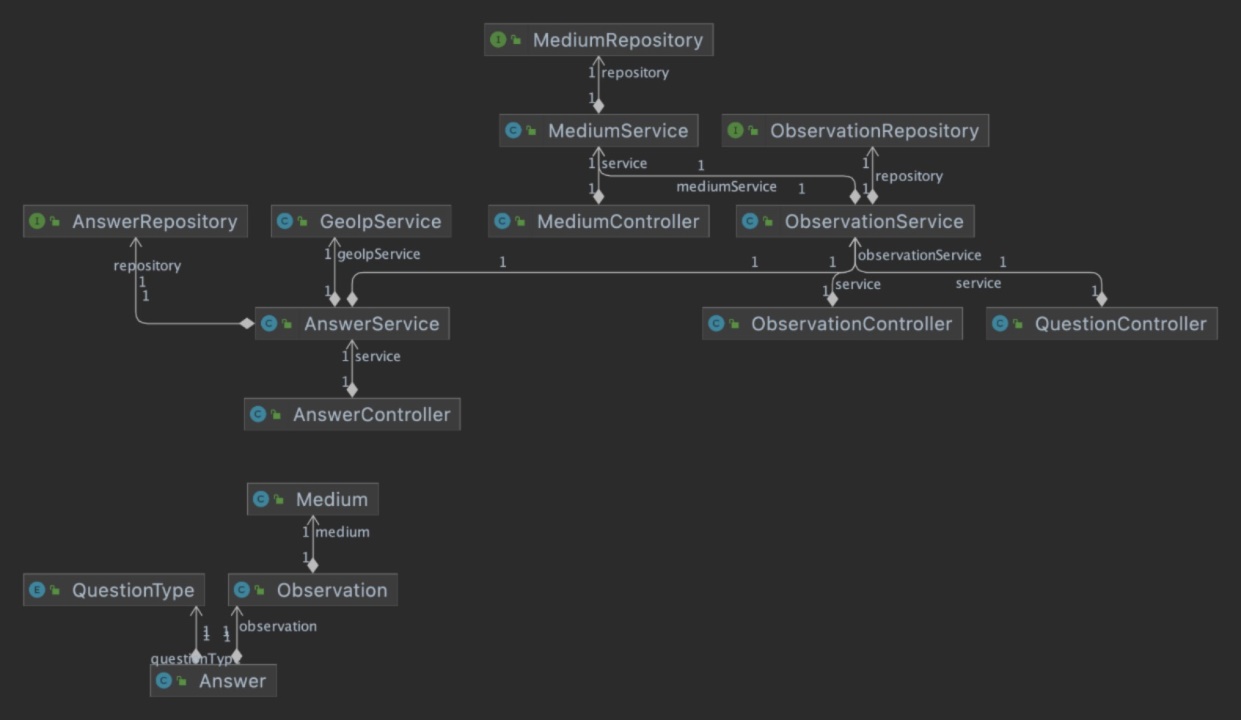

Supplement: baad003_Supp [file baad003_supp.zip › Appendices.docx]
